# COVID-19 pandemic partnership between medical students and isolated elders improves student understanding of older adults’ lived experience

**DOI:** 10.1186/s12877-022-03312-z

**Published:** 2022-08-02

**Authors:** Gray Moonen, Laure Perrier, Soumia Meiyappan, Sabrina Akhtar, Noah Crampton

**Affiliations:** 1 Bridgepoint Active Care, 1 Bridgepoint Dr, Toronto, ON M4M 2B5 Canada; 2 Sinai Health Systems, Toronto, Canada; 3 Department of Family and Community Medicine, Faculty of Medicine, University of Toronto, Toronto, Canada; 4 Toronto Western Family Health Team, University Health Network, Toronto, Canada; 5 Hospital for Sick Children, Toronto, Canada

**Keywords:** Aged, Loneliness, Well-being, Medical students, Cohort studies, COVID-19, Telephone, Communication

## Abstract

**Background:**

Evidence supports loneliness and social isolation as a strong risk factor for poor mental and physical health outcomes for older adults. The COVID-19 pandemic necessitated older adults isolate themselves for a prolonged duration. The Faculty of Medicine at the University of Toronto established the Student-Senior Isolation Prevention Partnership (SSIPP), a volunteer program involving telephone calls between medical students and older adults.

**Methods:**

A mixed methods pre-post study design included collecting quantitative data from older adults using the UCLA Loneliness Scale and the Warwick-Edinburgh Mental Well-being Scale. The study included 29 medical students and 47 older adults. The medical students filled out a questionnaire on self-perceived knowledge of social isolation, perception of seniors, attitudes towards seniors, and likelihood to engage in specialties focusing on older adults. Interviews were conducted with both the older adults and the medical students to understand each groups’ experiences and perspectives with taking part in the SSIPP program.

**Results:**

Participation in the program resulted in significant changes for medical students in areas such as increasing their likelihood to engage in care for older adults (*p* < .001), improving their knowledge of social isolation for seniors (*p* < .001), and the value of addressing social isolation in older adults (*p* < .001). The interviews conducted with the medical students support these findings and provide insight into contributing factors. Loneliness and mental well-being scales did not show significant changes for older adults however, our interviews revealed they considered the program to be valuable.

**Conclusions:**

Our results showed that after the communication outreach program, medical students’ perceptions were positively influenced towards older adults and they were more likely to pursue a career concentrated on older adults. The qualitative analysis revealed older adults valued the program. Timing and consistency of calls were factors identified by this group as having practical importance.

**Supplementary Information:**

The online version contains supplementary material available at 10.1186/s12877-022-03312-z.

## Background

Loneliness and social isolation in older adults is a growing public health concern. Over 40% of adults aged 60 or older report feeling lonely, while close to one-quarter of adults over 65 are considered to be socially isolated [1]. Loneliness is the “feeling of being alone, regardless of the amount of social contact”, and social isolation describes a lack of social connections [2]. There is strong evidence that older adults who are lonely or socially isolated have increased risks related to their health [1]. Loneliness is associated with increased risk of death, hospitalization, and emergency department visits for older adults with heart failure [1]. Social isolation is linked to an increased risk of premature mortality [3–6] and developing dementia [1]. This growing concern is coupled with research that suggests ageism amongst physicians-in-training is an issue that may impact social empathy and contribute to a lack of understanding older adults’ care needs [7–9]. To address this, the University of Toronto Faculty of Medicine established the Student-Senior Isolation Prevention Partnership (SSIPP), a volunteer program involving visits and calls between 170 medical students and over 300 older adults (www.ssipp.info) [10]. Programs linking seniors with medical students exist elsewhere with a common theme of providing an opportunity for medical students to develop an understanding of care while also providing emotional support to isolated older adults [11–14]. Office and colleagues [13] surveyed health science students who volunteered to make one phone call to an older adult living in long-term care facilities in the community who were at risk of isolation. Most students felt the calls were well-received by the participants and that making the phone call was positive. The older adults were appreciative of getting the phone call and of the volunteers’ time. Similarly, van Dyck and colleagues [14] reported on a telephone outreach program where medical students telephoned nursing home residents on a weekly basis. Both the volunteer students and the recreation directors, reporting on behalf of the residents at the nursing homes, provided positive reports about the program. Although methods for how data was collected for the study were not described, it was indicated that future work would involve collecting qualitative data from the student volunteers to report on the personal and professional impact of the program [14].

Governmental lockdowns during the COVID-19 pandemic increased the potential for isolation risk and focused attention on communication outreach programs. Given the increased interest in programs that connect students with older adults to address issues such as loneliness and social isolation, a rigorous evaluation is necessary to provide a better understanding of their impact. We planned a mixed methods study examining medical students and older adults who were matched to take part in regular phone or video calls. The purpose of the study is to determine the effect of regular medical student led outreach phone or video calls to older adults in the context of social isolation imposed by the COVID-19 pandemic.

## Methods

We used a concurrent triangulation mixed methods study design. The goal of this design is “to obtain different but complementary data on the same topic” [15] in order to understand a research issue. Collecting both quantitative (data that can be counted or measured) and qualitative data (descriptive or conceptual findings) identify the different types of data obtained. These are complementary as the information obtained provides a more refined and complete understanding of the topic [16]. Through the SSIPP program, medical students were paired with an older adult to engage in regular phone calls for a period of 12 weeks from May to August 2020. The students were instructed to allow older adults to speak on whatever they liked, and to focus on providing support through empathic listening. The planning and coordination of the calls such as timing (e.g., time of day, day of week, number of calls per week) or method of communication (e.g., video call, phone call) was left to the student and older adult to coordinate so that it would be agreeable and convenient to both parties. Calls were intended to be about 15–30 min in length. The UCLA Loneliness Scale [17] and the Warwick-Edinburgh Mental Well-being Scale [18] were administered pre- and post-intervention. As well, the Lubben Social Network Scale [19] was used prior to starting the program. The medical students were surveyed on knowledge of social isolation and attitudes towards older adults. Interviews were conducted with both medical students and older adults to offer a richer understanding of the impact and effects of this type of program. Medical students and older adults were recruited independently within the SSIPP program for our study and we made no attempt to recruit matched pairs, (i.e., collect data from both an older adult and the medical student who were put together). As a result, the number of participants for each group is not the same. It is also possible that a medical student may have been connected with more than one older adult.

### Study populations

#### Older adults

Older adults were identified by physicians from the Toronto Western Hospital Family Health Team. The Toronto Western Hospital Family Health Team is part of Toronto Western Hospital (twfht.ca), a University of Toronto teaching hospital within the University Health Network, and is an interdisciplinary primary health care practice that provides comprehensive family health care services to a catchment area in the city of Toronto, Canada. Participants were eligible if they were aged 70 years or older, English-speaking, and able to provide consent. Older adults were either emailed or mailed a consent form after agreeing to take part in the study and were contacted at a later date to obtain verbal consent over the phone. We focused on seniors aged 70 years or older as this group is identified as most vulnerable [20].

#### Medical students

The Student-Senior Isolation Prevention Partnership (SSIPP) program hosts a database listing of undergraduate medical students at the University of Toronto who are currently volunteering for the program. Emails were sent to student volunteers inviting them to take part in the study. After replying to the email, informed consent was obtained from the medical student and they were then matched with an older adult. The medical students contacted the older adult they had been matched with, and made arrangements for regular telephone calls.

### Instruments

#### Older adult questionnaire and validated scales

After obtaining informed consent, the older adults were asked to fill out a questionnaire that collected demographic information (Additional file 1) as well as three validated scales. The Lubben Social Network Scale (Additional file 1) is a validated six-point scale used to assess social isolation in older adults by measuring perceived social support received by family and friends [19]. This was used pre-intervention to understand the extent of each person’s isolation. We measured loneliness with the UCLA Loneliness Scale [16] that consists of 20 items (Additional file 2). Responses are provided on a 4-point Likert scale from ‘often’ to ‘never’. The UCLA Loneliness Scale is a validated scale designed to measure subjective feelings of loneliness as well as feelings of social isolation [17]. Finally, the Warwick-Edinburgh Mental Well-being Scale [18] (Additional file 2), a 14-item scale rated on a 5-point scale ranging from ‘none of the time’ to ‘all of the time’, was filled out prior to receiving phone calls from the medical students, as well as post-intervention. The Warwick-Edinburgh Mental Well-being Scale is a “measure of mental well-being focusing entirely on positive aspects of mental health” [18].

#### Medical student questionnaires

Prior to placing phone calls to the older adults, the medical students filled out a demographic questionnaire (Additional file 3). As well, they completed a 5-item questionnaire on knowledge of social isolation, perception of seniors, attitudes towards seniors, and likelihood to engage in specialties focusing on older adults that was developed based on the Semantic Differential Technique (Additional file 4) [21] and was administered at the 12-week point in a retrospective post-then-pre method [22]. Each item was rated on a 5-point scale from 1 (weak) to 5 (strong).

#### Interviews

An interview guide was developed for the older adults (Additional file 5) and the medical students (Additional file 6) that asked participants about their experience and perspectives of taking part in the communication outreach program. Interviews were conducted by a member of the study team for both medical students and older adults between June 2020 and August 2020 post-intervention.

### Data analysis

Data was collected by telephone for all questionnaires and interviews for both older adults and medical students. Analysis was conducted on the medical student’s questionnaire data with descriptive statistics and a paired t-test (two-tailed) was used to analyze the difference between baseline and post-intervention measures. A *p*-value of < 0.05 was considered statistically significant. Participants with missing scores were excluded from the statistical analysis. Interview recordings were transcribed verbatim by a transcriptionist. For both the medical students and the older adult transcripts, two investigators (GM, LP, NC) independently generated codes by reading through all transcripts, reflecting on the interview guide, and then independently coding an initial transcript. A meeting was held to compare codes, discuss commonalities, and harmonize inconsistencies. The codes were refined through consensus and included collapsing and adding codes to more accurately represent the data until a final set of codes was generated. After this, two transcripts were coded independently as a set by two researchers and a meeting was held afterwards in order to review coding, resolve discrepancies, and refine the set of codes. Once coding was completed, data was reviewed to create themes and sub-themes within the codes. Microsoft Excel (Microsoft Corp., USA) was used to organize data by codes, and arrange by themes and sub-themes. Lincoln and Guba’s [23] framework was used to enhance the rigour and quality of the study. Thematic saturation was determined using the method validated by Guest and colleagues [24] (Additional file 7). Quotes from focus group participants are provided for transparency that offer support for themes and allow readers to judge whether the findings reflect the perceptions of participants. An audit trail of coding and theme development was generated by documenting procedures; this process of triangulation ensured that findings arose from consensus amongst the investigators. The study was approved by the University Health Network Research Ethics Board.

## Results

### Older adult questionnaire and validated scales

A total of 47 participants completed the study (Table 1) and seven participants were lost to follow up. No significant difference pre- and post-intervention was found for both the UCLA Loneliness Scale and the Warwick-Edinburgh Mental Well-being Scale (Table 2). Upon analyzing the older adult sub-group who scored moderately or high risk on the UCLA Loneliness Scale in the pre-intervention questionnaire (total of 11 participants), There is an improvement in scores, from pre-intervention (mean 58.64, SD 8.23) and post-intervention (mean 53.91, SD 3.37); t_10_ = 2.13, *p* = 0.06. While it did not reach statistical significance in our sample, there may be a trend toward a true effect given the *p*-value of 0.06, just above the threshold of 0.05. Scores for both scales pre- and post-intervention are reported in Additional file 9 and indicate that prior to taking part in the program, the majority of older adults were not high risk for loneliness (36/47, 77%) and scored high-to-average for mental well-being (42/47, 89%). The data collected on the Lubben Social Network Scale indicated the majority of participants (37/47, 79%) had good social networks which includes connections to family and friends (Additional file 8).

**Table 1:** Characteristics of Study Population

**Older Adults**	**Number (%**^a^**)**	**Medical Students**	**Number (%**^a^**)**
Total Number	47	Total Number	29
Age		Age	
65–69	1 (2)	22–26	25 (86)
70–79	33 (70)	27–34	4 (14)
80–89	10 (21)	Sex	
90 +	3 (6)	Female	19 (66)
Sex		Male	10 (35)
Female	32 (68)	Year of Medical School	
Male	15 (32)	1st	10 (35)
Racial/ethnic group		2nd	9 (31)
Asian – East	1 (2)	3rd	8 (28)
Asian – South	0 (0)	4th	2 (7)
Asian – South East	2 (4)	Medical field of interest^b^	
Black—African	0 (0)	Family medicine	21
Black-Caribbean	1 (2)	Internal medicine	15
Black-North American	0 (0)	Geriatrics	8
First Nations	0 (0)	Pediatrics	6
Indian-Caribbean	0 (0)	Neurosurgery/Neurology	0
Indigenous/Aboriginal	0 (0)	Dermatology	2
Inuit	0 (0)	Psychiatry	5
Latin American	1 (2)	General Surgery	3
Métis	0 (0)	Ophthalmology	0
Middle Eastern	0 (0)	Immunology	1
White-European	25 (53)	Radiology	1
White-North American	15 (32)	Orthopedics	2
Mixed heritage	1 (2)	Obstetrics/Gynecology	5
Other	0 (0)	Palliative Care	4
Do not know	0 (0)	Urology	2
Prefer not to answer	1 (2)	Endocrinology	3
Education		Other	0
No formal education	1 (2)	Career focus on older adults	
High school	9 (19)	Yes	23 (82)
College	11 (23)	No	5 (18)
University	9 (19)		
Masters	9 (19)		
Doctorate	6 (13)		
Other	2 (4)		
Marital status			
Single	7 (15)		
Married	23 (49)		
Common-law	0 (0)		
Divorced	3 (6)		
Widowed	14 (30)		
Prefer not to answer	0 (0)		

^a^ Numbers may not equal 100 due to rounding

^b^ More than one medical field could be chosen

**Table 2:** UCLA Loneliness and Warwick-Edinburgh Mental Well-being Scales Pre- and Post-Intervention

	**mean (SD)**	**mean (SD)**	**mean difference (95% CI)**	**t-test (df)**	***p*-value**
	**Pre-intervention** ***N* = 47**	**Post-intervention** ***N* = 47**			
UCLA Loneliness Scale	39.11 (2.06)	38.83 (1.98)	‒0.28 (‒0.55‒1.11)	0.21 (46)	0.83
Warwick-Edinburgh Mental Well-being Scale	52.68 (1.13)	52.51 (1.26)	‒0.17 (‒0.32‒0.66)	2.13 (46)	0.85

### Older adult interviews

We conducted interviews with six older adults. We identified eight themes from the interviews and these were agreeableness, connectedness, memorable substance of calls, recognition of value, self concept, effect of COVID-19 pandemic, mutually beneficial, and consistency. Each theme is described below and illustrative quotes are summarized in Table 3.

**Table 3:** Themes and illustrative quotes for older adults

Theme	Illustrative quote
Agreeableness	…just a lovely individual. She was warm. She brightened my day. (Participant #18)
	…she is very pleasant…. She is easy to talk with….and it comes down on the positives. Very much on the positive. (Participant #33)
Connectedness	She will ask questions that have continuity to them. Like she knows that I have a dog. So, she will ask me you know how my dog is doing. So, she obviously makes notes so that she could bring up…points of conversation that we can carry on exchanging. It is not just her listening to me whine. (Participants #33)
	I got a feeling that it was like a friend that I had known for quite some time who was able to empathize with the kinds of things that I was going through…there was somebody out there that was throwing me a lifeline for an hour a week enabled me to, to, to, um, accept this isolation. (Participant #18)
Memorable substance of calls	…and I want to hear more next week when we chit chat. I want to hear more about that about her visit back home. I will be quizzing her. (Participant #43)
	…we talk about her running and what I am doing, out walking my dog. (Participant #33)
Recognition of value	I knew that there was someone there if, you know, if everything else failed. (Participant #91)
	And that person would not know the extent to which she did help me… I wouldn't have the language skills to be able to choose the words to be able to explain how much I look forward to that hour or so. (Participant #18)
	….it has very positive value for people like myself. (Participant #43)
Self-concept	I am quite a social person. I think everybody gets a little lonely sometimes in their own way. (Participant #91)
	…and I said oh no I am I am a perennial optimist. I wouldn't go down that road. You know…to a point where I wouldn't be able to come up, I am not that kind of person. (Participant #11)
Effect of COVID-19 pandemic	I am a two-time cancer survivor. I did not know what the virus would do, but I did know that I had to stay inside as much as I could….I kind of thought to myself how am I going to handle this. (Participant #18)
Mutually beneficial	I think that the medical student who will one day become the doctor and they will have a greater empathy for patients. And particularly for older patients. (Participant #18)
	…these…young people. Interesting to see have they grew over three or four weeks. (Participant #91)
Consistency	The second guy I had…about three weeks ago I missed his call on a Monday… and he never phoned again. So that one didn't work out…. (Participant #91)
	…she could have let me know that she is not going to be around, you know? (Participant #33)

#### Agreeableness

The theme, agreeableness, addresses older adult’s perspectives on the medical students they were paired with. Complimentary terms were used including warm, personable, easy to talk with, and polite. Being positive and upbeat were qualities that were welcomed by the older adults in their interactions with their student partners.

#### Connectedness

Connectedness emerged as a theme where the older adults described feeling that they had made a new friend, and that the student partner contributed to diminished feelings of isolation. Bonds were cultivated and this was signaled by the continuity of conversation from one week to the next over common areas of interest that were not necessarily related to their health status. When connections faltered, inconsistent communication (e.g. student forgetting to call, or calling on a different day) was a factor.

#### Memorable substance of calls

This theme describes the mutual sharing of diverse interests, experiences, and perspectives that enriched the conversations between the older adults and the student partners. Older adults discussed a wide range of subjects from everyday things such as books and pets, as well as identifying very specific areas of common interest. Topics also included checking on the older adults’ health and well-being.

#### Recognition of value

The recognition of value theme summarizes the older adults’ gratitude for the program and includes their overall feelings of appreciation. Sentiments included wishing the program would not end, indicating they would recommend the program to their peers, and indicating they hoped the program continued.

#### Self concept

The theme, self concept, captures how older adults perceived their own nature and state of mind toward their current circumstances. Living alone and acknowledging that isolation was an issue identified by participants.

#### Effect of COVID-19 pandemic

COVID-19 altered the normal routine of lives and this theme encompasses how the pandemic affected the lives of the older adults. This included how their personal circumstances made them more vulnerable due to illness, or not having family members nearby.

#### Mutually beneficial

This theme describes older adults’ impressions of how the program may have had benefits for the medical students and included recommendations for making it a part of the regular medical school curriculum.

#### Consistency

The consistency theme highlights the importance the older adults placed on having the same student partner each week, and ensuring the conversations occurred on the same day and time each week. When this consistency was missing, the older adults' experience of the program was negatively affected.

### Medical student questionnaires

Twenty nine medical students completed a demographic questionnaire (Table 1). None were lost to follow up. They also completed a questionnaire on knowledge of social isolation and attitudes towards seniors at the 12-week point in a retrospective post-then-pre method. All questions showed statistically significant results. Each question is summarized in Table 4.

**Table 4:** Medical student questionnaire: Mean and (SD) pre-intervention and post-intervention

	**mean (SD)**	**mean (SD)**	**mean difference (95% CI)**	***p*-value**
	**Pre-intervention** ***N* = 29**	**Post-intervention** ***N* = 29**		
Your knowledge of social isolation	3.10 (0.56)	3.97 (0.57)	0.86 (-0.56–1.16)	.001
Your understanding of the value in addressing social isolation among older adults	3.34 (0.77)	4.48 (0.51)	1.14 (-0.80–1.48)	.001
Your attitude towards seniors	4.07 (0.70)	4.52 (0.51)	0.45 (-0.13–0.77)	.001
Your likelihood to engage in care with older adults	3.76 (0.95)	4.34 (0.77)	0.59 (-0.13–1.05)	.001
The priority you put on addressing social isolation among older adults	3.07 (0.70)	4.34 (0.55)	1.27 (-0.94–1.60)	.001

### Medical student interviews

We conducted interviews with five medical students. The seven themes that were identified from the interviews were 1) fulfilling, 2) impactful, 2) valuable opportunity, 3) connectedness, 4) empathy, 5) COVID-19 isolation, 6) career influence, and 7) barriers to care. Each theme is described below and illustrative quotes are summarized in Table 5.

**Table 5:** Themes and illustrative quotes for medical students

Theme	Illustrative quote
Fulfilling	…she will open the conversation with oh it is so lovely to hear your cheerful voice. And I think that brightens my day as well. So, I have had a really positive experience. (Participant #03)
	… it was really positive experience. I felt like it was truly beneficial, and it was a nice way to meet somebody new. (Participant #41)
Impactful	But I think it kind of highlighted for me… how much of a difference…a little check-in phone call can make in someone's day. (Participant #03) … it is not a huge time commitment, but… you are still making some difference in the lives of the people that you are calling every week. You know like it only takes like 15 to 20 min (Participant #14) It is just like a small, little bit of energy and time on my end. I think can make a really positive difference in folks’ lives (Participant #42
Valuable opportunity	… I am actually already seeing it impact my medical practice because…I didn't really truly appreciate the huge impact that social isolation can have. (Participant #09)
	It is a good opportunity to help somebody else. But I think you also feel like really good about it. And you can feel like you are making a positive impact. (Participant #42)
Connectedness	…I think it is good practice for building rapport with someone, like building their trust and developing our relationship (Participant #11)
	I think it has been nice for myself and also just for the patient to have this weekly the check in. And see how each of our weeks have been doing. (Participant #11)
Empathy	I notice that I am actively asking questions relating to social isolation. And trying to strategize ways to help seniors alleviate the social isolation in their lives (Participant #09)
	… just the importance of you know keeping in touch with people who are more prone to isolation. (Participant #14)
COVID-19 isolation	…social isolation is a pretty big problem pre-COVID and will continue to be one post-COVID. (Participant #42)
	… One thing is how big of an impact social isolation has on health outcomes in the elderly. And I learned important strategies for how to alleviate the social anxiety and the social isolation during a pandemic setting. (Participant #09)
Career influence	…with this program I am realizing that that [Geriatrics] is an area that I didn't think that I would be interested in. (Participant #41) You know I have always loved to work with older adults but it kind of reinforces…I definitely want to work with them in the future. (Participant #14)

#### Fulfilling

The theme, fulfilling, illustrates the medical students’ perspectives on taking part in the program and making the phone calls to the older adults. Positive terms were used including rewarding and enjoyable.

#### Impactful

The impactful theme describes the effect the students felt they were having on the older adult’s life and includes descriptions of the ways they believed they had made a contribution. For example, providing reliable information that countered confusing media reports.

#### Valuable opportunity

This theme highlights the perception amongst students that this was a unique opportunity that reflected the difference between formal learning and experiences outside of the classroom or clinical setting.

#### Connectedness

Connectedness emerged as a theme where the students described looking forward to interacting with the older adults, as well as having a sincere interest in the person and in engaging with them.

#### Empathy

The empathy theme described how this learning opportunity offered a more fulsome understanding of older adults’ experiences and contributed to an understanding of unique issues seniors faced during the COVID-19 pandemic.

#### COVID-19 isolation

This theme highlighted the student’s understanding that isolation was an issue that pre-dated COVID-19, but that the pandemic has made it worse.

#### Career influence

The career influence theme describes a new or reinforced interest in caring for older adults or pursuing fields that include the care of older adults. 

## Discussion

Our study presents evidence that the SSIPP program improved medical students’ perspectives on caring for older adults. The results were statistically significant and indicate that participation in this program was associated with strengthening knowledge of social isolation, attitude toward seniors, likelihood to engage in caring for older adults, understanding of the value in addressing social isolation among older adults, and the priority the students put on addressing social isolation. Our qualitative analysis of the medical students’ interviews provide further support that the experience had impact on influencing their careers and their current clinical practice. Medical students described actively asking more questions about social isolation and recognizing that social isolation would continue to be a problem post-COVID-19 for seniors. As well, the medical students perceived minimal effort was required for the phone call check-ins and indicated benefit to both their own sense of fulfillment and the older adult they were paired with was substantial. For older adults, the scores from the UCLA Loneliness Scale and the Warwick-Edinburgh Mental Well-being Scale did not change significantly over the intervention period. However, analysis of the interview data provides insights that the older adults genuinely looked forward to the phone calls and if a call was missed, it was described as disappointing. This finding is useful to planners in emphasizing the importance of regularity in making phone calls to older adults, for both day of the week and time of day, when recruiting medical students. It also reveals that the once-a-week phone call had benefit for this group, with factors that need further investigation.

### Strengths and limitations of the study

Our mixed methods study combined quantitative data with qualitative interviews. The strength of this approach was that the outcomes reported in our quantitative analysis could be explained and contextualized with our qualitative data. For the medical students, the quantitative data showed a shift in perspectives and qualitatively offered insightful understanding to the interactions with seniors, including details of how it influenced their perspectives on their career and dealing with seniors in their medical practice. For the older adults, the qualitative data provided support that the program was beneficial to the participants. Our quantitative data did not show a change in loneliness or well-being through the use of our validated scales and this identifies a limitation of our study. Our group of seniors self-selected into the program and the people who chose to participate had scores for both the UCLA Loneliness Scale and the Warwick-Edinburgh Mental Well-being Scale that were not of concern for both loneliness and well-being. For the UCLA Loneliness Scale, the majority of older adults (36/47, 77%) were not considered at risk for loneliness. For the Warwick-Edinburgh Mental Well-being Scale, 89% (42/47) of the seniors scored as having average-to-high mental well-being and not considered at risk as well. This made it difficult to determine if our program has impact on loneliness and well-being. However, our qualitative analysis of the interviews with the older adults provides rich information that the program had value for them. Our qualitative themes highlight this and includes participants stating how important calls were to them (Recognition of Value theme), to being unhappy and disappointed when the calls were missed (Consistency theme). It is interesting to note that although some content of the calls were about health, such as helping to decipher media items related to COVID-19, the conversations were about everyday matters such as pets, neighbourhoods, and personal experiences. Some older adults commented that they were pleased at the opportunity to speak to someone much younger than themselves so that conversations were not about ‘aches and pains’. We had a small sample size and with the older adults, seven participants were lost to follow up. As well, the older adults were largely white (40/47, 85%) and had higher levels of education, with the majority holding a college or university degree (35/47, 74%). This reflects the recruitment strategy of seeking volunteers as participants rather than screening for specific participant characteristics.

### Directions for future research

The results of this study are promising and suggest future studies would provide more insight on communication outreach programs for both older adults and medical students. Future research could look at identifying high-risk seniors during study recruitment to determine if a communication outreach program has a quantitative impact for factors such as loneliness or mental well-being. As well, it would be helpful to determine the effect of factors such as the length of intervention, or different COVID-19 lock-down levels, (e.g., broad allowance of activities, or restriction/closure of most businesses/organizations).

## Conclusions

We found significant results that after the SSIPP partnership program, medical students’ perspectives were positively influenced and they were more likely to engage in a medical career focused on older adults. With an aging population, it is important to identify factors that contribute to optimal well-being. The older adults in our study indicated they found the communication outreach program valuable in our qualitative analysis and identified features that they considered of practical importance, such as the timing and consistency of calls. We did not find significant reductions in loneliness or mental well-being in older adults since the majority of our self-selected cohort were not at risk for either factor.

## Supplementary Information

**Additional file 1. **Demographic questionnaire – Older adult participants. *Question 6 is the Lubben Social Network Scale.

**Additional file 2. **UCLA Loneliness Scale and Warwick-Edinburgh Mental Well-being Scale.

**Additional file 3. **Demographic questionnaire – Medical student participants.

**Additional file 4. **Questionnaire for Medical Student Participants.

**Additional file 5. **Interview guide – Older adult participants.

**Additional file 6. **Interview guide – Medical student participants.

**Additional file 7. **Thematic saturation of themes.

**Additional file 8. **Lubben Social Network Scale Scores.

**Additional file 9. **UCLA Loneliness Scale and Warwick-Edinburgh Mental Well-being Scale Scores for Older Adults.

## Data Availability

The datasets used and/or analyzed during the current study are available from the corresponding author on reasonable request.

## Abbreviation

SSIPP: Student-Senior Isolation Prevention Partnership
