# Supplementary material for: COVID-19 pandemic partnership between medical students and isolated elders improves student understanding of older adults’ lived experience
Source: BMC Geriatr. 2022 Aug 2;22:636. doi: 10.1186/s12877-022-03312-z (PMC9344259; doi:10.1186/s12877-022-03312-z)
Supplement: Supplementary file 1 — Additional file 1. Demographic questionnaire – Older adult participants. *Question 6 is the Lubben Social Network Scale. [file 12877_2022_3312_MOESM1_ESM.docx]

**Additional file 1.** Demographic questionnaire – Older adult participants
*Question 6 is the Lubben Social Network Scale

Participant ID: _________________

Date: ________________________

**1. What is your age?** ____________ (years)

⬜ 70-74 ⬜ 75-79 ⬜ 80-84 ⬜ 85-89 ⬜ 90+

**2. What is your gender?** (check one)

⬜ Male ⬜ Female ⬜ Prefer not to answer ⬜ Other (please specify): ___________

**3. Which of the following best describe(s) your racial or ethnic group?** (check all that apply)

⬜ Asian - East (e.g. Chinese, Japanese, Korean)

⬜ Asian - South (e.g. Indian, Pakistani, Sri Lankan)

⬜ Asian - South East (e.g. Malaysian, Filipino, Vietnamese)
⬜ Black - African (e.g. Ghanaian, Kenyan, Somali)
⬜ Black - Caribbean (e.g. Barbadian, Jamaican)
⬜ Black - North American (e.g. Canadian, American)
⬜ First Nations American) Please specify: ______________________
⬜ Indian - Caribbean (e.g. Guyanese with origins in India)
⬜ Indigenous/Aboriginal - not included elsewhere
⬜ Inuit
⬜ Latin American (e.g. Argentinean, Chilean, Salvadoran)
⬜ Métis

⬜ Middle Eastern (e.g. Egyptian, Iranian, Lebanese)

⬜ White - European (e.g. English, Italian, Portuguese, Russian)

⬜ White - North American (e.g. Canadian, American)

⬜ Mixed heritage (e.g. Black - African & White – North)

⬜ Other(s): Please specify:______________________

⬜ Do not know

⬜ Prefer not to answer

**4. What is the highest level of education you have completed?** (check one)

⬜ No formal education ⬜ High School ⬜ College ⬜ University ⬜ Masters

⬜ Doctorate (e.g. PhD, MD) ⬜ Other, please specify ___________________

**5. What is your marital status?** (check one)

⬜ Single ⬜ Married ⬜ Common-Law ⬜ Divorced ⬜ Widowed

⬜ Prefer not to answer

**6. Please answer the following questions about your social network by indicating your response with an X under the column that applies.***

|  | **Score** | | | | | |
| --- | --- | --- | --- | --- | --- | --- |
|  | **0** | **1** | **2** | **3** | **4** | **5** |
| How many relatives do you see or hear from at least once a month? |  |  |  |  |  |  |
| How many relatives do you feel at ease with that you can talk about private matters? |  |  |  |  |  |  |
| How many relatives do you feel close to such that you could call on them for help? |  |  |  |  |  |  |
| How many friends do you see or hear from at least once a month? |  |  |  |  |  |  |
| How many friends do you feel at ease with that you can talk about private matters? |  |  |  |  |  |  |
| How many friends do you feel close to such that you could call on them for help? |  |  |  |  |  |  |

**7. What are the first three digits of your postal code?** ___________________
