# Supplementary material for: COVID-19 pandemic partnership between medical students and isolated elders improves student understanding of older adults’ lived experience
Source: BMC Geriatr. 2022 Aug 2;22:636. doi: 10.1186/s12877-022-03312-z (PMC9344259; doi:10.1186/s12877-022-03312-z)
Supplement: Supplementary file 2 — Additional file 2. UCLA Loneliness Scale and Warwick-Edinburgh Mental Well-being Scale. [file 12877_2022_3312_MOESM2_ESM.docx]

**Additional file 2.** UCLA Loneliness Scale and Warwick-Edinburgh Mental Well-being Scale

Participant ID: _________________

Date: ________________________

- Participant agrees to attempt to refrain from using personal health information such as names and family members

1. **Please take a look at the following items and indicate your response with an X in the appropriate column**

| **Item** | **Response** | | | |
| --- | --- | --- | --- | --- |
|  | **Often** | **Sometimes** | **Rarely** | **Never** |
| 1. I am unhappy doing so many things alone. |  |  |  |  |
| 2. I have nobody to talk to. |  |  |  |  |
| 3. I cannot tolerate being so alone. |  |  |  |  |
| 4. I lack companionship. |  |  |  |  |
| 5. I feel as if nobody really understands me. |  |  |  |  |
| 6. I find myself waiting for people to call or write. |  |  |  |  |
| 7. There is no one I can turn to. |  |  |  |  |
| 8. I am no longer close to anyone. |  |  |  |  |
| 9. My interests and ideas are not shared by those around me. |  |  |  |  |
| 10. I feel left out. |  |  |  |  |
| 11. I feel completely alone. |  |  |  |  |
| 12. I am unable to reach out and communicate with those around me. |  |  |  |  |
| 13. My social relationships are superficial. |  |  |  |  |
| 14. I feel starved for company. |  |  |  |  |
| 15. No one really knows me well. |  |  |  |  |
| 16. I feel isolated from others. |  |  |  |  |
| 17. I am unhappy being so withdrawn. |  |  |  |  |
| 18. It is difficult for me to make friends. |  |  |  |  |
| 19. I feel shut out and excluded by others. |  |  |  |  |
| 20. People are around me but not with me. |  |  |  |  |

1. **Please take a look at the following items and indicate your response with an X in the appropriate column**

| **Item** | **Response** | | | | |
| --- | --- | --- | --- | --- | --- |
|  | **None of the Time** | **Rarely** | **Some of the Time** | **Often** | **All of the Time** |
| I’ve been feeling optimistic about the future |  |  |  |  |  |
| I’ve been feeling useful |  |  |  |  |  |
| I’ve been feeling relaxed |  |  |  |  |  |
| I’ve been feeling interested in other people |  |  |  |  |  |
| I’ve had energy to spare |  |  |  |  |  |
| I’ve been dealing with problems well |  |  |  |  |  |
| I’ve been thinking clearly |  |  |  |  |  |
| I’ve been feeling good about myself |  |  |  |  |  |
| I’ve been feeling close to other people |  |  |  |  |  |
| I’ve been feeling confident |  |  |  |  |  |
| I’ve been able to make up my own mind about things |  |  |  |  |  |
| I’ve been feeling loved |  |  |  |  |  |
| I’ve been interested in new things |  |  |  |  |  |
| I’ve been feeling cheerful |  |  |  |  |  |
