# Supplementary material for: COVID-19 pandemic partnership between medical students and isolated elders improves student understanding of older adults’ lived experience
Source: BMC Geriatr. 2022 Aug 2;22:636. doi: 10.1186/s12877-022-03312-z (PMC9344259; doi:10.1186/s12877-022-03312-z)
Supplement: Supplementary file 3 — Additional file 3. Demographic questionnaire – Medical student participants. [file 12877_2022_3312_MOESM3_ESM.docx]

**Additional file 3.** Demographic questionnaire – Medical student participants

Participant ID: _________________

Date: ________________________

**1. Which year of medical school are you currently in?**

⬜ 1^st^ ⬜ 2^nd^ ⬜ 3^rd^ ⬜ 4^th^ ⬜ Other (please specify): ___________

**2. What is your gender?** (check one)

⬜ Male ⬜ Female ⬜ Prefer not to answer ⬜ Other (please specify): ___________

**2. What is your age range?** (check one)

⬜ <22 ⬜ 22-26 ⬜ 27-34 ⬜ 35-44 ⬜ >44

**3. Which of the following best describe(s) your medical field(s) of interest?** (check all that apply)

⬜ Family Medicine ⬜ Internal Medicine ⬜ Geriatrics ⬜ Pediatrics

⬜ Neurosurgery/Neurology ⬜ Dermatology ⬜ Psychiatry ⬜ General Surgery

⬜ Ophthalmology ⬜ Immunology ⬜ Radiology ⬜ Orthopedics

⬜ Obstetrics/ Gynecology ⬜ Palliative Care ⬜ Urology ⬜ Endocrinology

⬜ Other (please specify): __________________________

**4. Would you consider a career focused on interacting with older adults (e.g. geriatrics, internal medicine, palliative care)?**

⬜ Yes ⬜ No
