# Supplementary material for: COVID-19 pandemic partnership between medical students and isolated elders improves student understanding of older adults’ lived experience
Source: BMC Geriatr. 2022 Aug 2;22:636. doi: 10.1186/s12877-022-03312-z (PMC9344259; doi:10.1186/s12877-022-03312-z)
Supplement: Supplementary file 4 — Additional file 4. Questionnaire for Medical Student Participants. [file 12877_2022_3312_MOESM4_ESM.docx]

**Additional file 4.** Questionnaire for Medical Student Participants

Participant ID: _________________

Date: ________________________

***Pre-SSIPP Program***

Now that you have completed the SSIPP Program (regular phone calls with an older adult), how would you rate the following ***BEFORE*** this program? (1 = weak, 5 = strong). Please mark with an “X” under the column that denotes your rating.

| **Item** | **Rating** | | | | |
| --- | --- | --- | --- | --- | --- |
|  | **1** | **2** | **3** | **4** | **5** |
| Your knowledge of social isolation |  |  |  |  |  |
| Your understanding of the value in addressing social isolation among older adults |  |  |  |  |  |
| Your attitude towards seniors |  |  |  |  |  |
| Your likelihood to engage in care with older adults |  |  |  |  |  |
| The priority you put on addressing social isolation among older adults |  |  |  |  |  |

***Post-SSIPP Program***

Now that you have completed the SSIPP Program (regular phone calls with an older adult), how would you rate the following ***AFTER*** this intervention? (1 = weak, 5 = strong). Please mark with an “X” under the column that denotes your rating.

| **Item** | **Rating** | | | | |
| --- | --- | --- | --- | --- | --- |
|  | **1** | **2** | **3** | **4** | **5** |
| Your knowledge of social isolation |  |  |  |  |  |
| Your understanding of the value in addressing social isolation among older adults |  |  |  |  |  |
| Your attitude towards seniors |  |  |  |  |  |
| Your likelihood to engage in care with older adults |  |  |  |  |  |
| The priority you put on addressing social isolation among older adults |  |  |  |  |  |

Please answer the following questions:

1. How would you describe your overall experience in the SSIPP Program?
2. How would you describe your attitude towards older adults?
3. Please describe any particularly impactful experiences you had during the SSIPP program.
4. Would you recommend this program to other medical students? (circle one)

⬜ Yes ⬜ No

- 1. If yes, what is one thing you would tell a future medical student about this program?
  2. If no, what is one thing you would change about this program?
