# Supplementary material for: COVID-19 pandemic partnership between medical students and isolated elders improves student understanding of older adults’ lived experience
Source: BMC Geriatr. 2022 Aug 2;22:636. doi: 10.1186/s12877-022-03312-z (PMC9344259; doi:10.1186/s12877-022-03312-z)
Supplement: Supplementary file 5 — Additional file 5. Interview guide – Older adult participants. [file 12877_2022_3312_MOESM5_ESM.docx]

**Additional file 5.** Interview guide – Older adult participants

Participant ID: _________________

Date: ________________________

Initials of Interviewer: __________

- Participant agrees to attempt to refrain from using personal health information such as names and family members

1. How did the phone call outreach program affect you?
   1. *Prompt: What has it meant to you to have a volunteer?*
2. What kinds of things did you talk about? What activities did you do?
3. What were some of the things you liked that volunteers did? What were some of the things that you would prefer the volunteers wouldn’t do?
4. Were there any notable experiences that stood out?
   1. *Prompt: What was the best part of your experience?*
   2. *Prompt: What was the worst part? Is there anything that you would change?*
5. Did you feel lonely or socially isolated before this program? Has this experience improved those feelings at all?
6. Do you feel this program has helped you feel more connected to others?
   1. *Prompt: Has this program provided companionship?*
   2. *Prompt: Has this program improved your feelings of loneliness from before the program?*
7. Would you participate in the program again or recommend it to other older adults?
