# Supplementary material for: COVID-19 pandemic partnership between medical students and isolated elders improves student understanding of older adults’ lived experience
Source: BMC Geriatr. 2022 Aug 2;22:636. doi: 10.1186/s12877-022-03312-z (PMC9344259; doi:10.1186/s12877-022-03312-z)
Supplement: Supplementary file 6 — Additional file 6. Interview guide – Medical student participants. [file 12877_2022_3312_MOESM6_ESM.docx]

**Additional file 6.** Interview guide – Medical student participants

Participant ID: _________________

Date: ________________________

Initials of Interviewer: __________

1. Tell me more about your experience in the SSIPP program.
   - *Prompt: Please describe your experience with older adults in the SSIPP program.*
2. What were some of the key learnings you took away from this program?
   - *Prompt: What is your understanding of social isolation?*
3. Please describe any particularly impactful experiences during the SSIPP program.
4. How might your participation in the SSIPP program influence your medical practice in the future?
   - *Prompt: Do you feel you are more likely to work with older adults in the future*
5. What is one thing you would tell a future medical student about this program?
