# Supplementary material for: COVID-19 pandemic partnership between medical students and isolated elders improves student understanding of older adults’ lived experience
Source: BMC Geriatr. 2022 Aug 2;22:636. doi: 10.1186/s12877-022-03312-z (PMC9344259; doi:10.1186/s12877-022-03312-z)
Supplement: Supplementary file 7 — Additional file 7. Thematic saturation of themes. [file 12877_2022_3312_MOESM7_ESM.docx]

**Additional file 7.** Thematic saturation of themes

**Older Adults**

| **Date \| Coding Agreement** | **Transcript(s)** |  |
| --- | --- | --- |
| 21 Jan 2021 | Interview #5 \| Interview #11 | *6 original codes agreed upon* |
| 28 Jan 2021 | Interview #18 | 2 new codes added:  1) Mutually Beneficial;  2) Consistency |
| 3 Feb 2021 | Interview #43 | No new codes added |
| 10 Feb 2021 | Interview #33 | No new codes added |
| 18 Feb 2021 | Interview #91 | No new codes added |

**Saturation calculation:**

| **Interview number** | **1** | **2** | **3** | **4** | **5** | **6** |
| --- | --- | --- | --- | --- | --- | --- |
| **New themes per interview** | 6 | 0 | 2 | 0 | 0 | 0 |
| **# Base themes** |  | 6 | 2 (33%) | 0 (0%) | 0 (0%) | 0 (0%) |

The fourth to sixth interview quotient of 0% is below the ≤ 5% threshold. The amount of new information has diminished to a level where saturation has been reach based on this measure.

**Medical Students**

| **Date \| Coding Agreement** | **Transcript(s)** |  |
| --- | --- | --- |
| 27 Jan 2021 | Interview #3 \| Interview #14 | *7 original codes agreed upon* |
| 03 Feb 2021 | Interview #9 | 1 new code added:  Barriers to Care |
|  | Interview #41 | No new codes added |
|  | Interview #42 | No new codes added |

**Saturation calculation:**

| **Interview number** | **1** | **2** | **3** | **4** | **5** |
| --- | --- | --- | --- | --- | --- |
| **New themes per interview** | 7 | 0 | 1 |  |  |
| **# Base themes** |  | 7 | 1 (14%) | 0 (0%) | 0 (0%) |

The fourth and fifth interview quotient of 0% is below the ≤ 5% threshold. The amount of new information has diminished to a level where saturation has been reach based on this measure.

Source: Guest G, Namey E, Chen M. A simple method to assess and report thematic saturation in qualitative research. PLoS One. 2020 May 5;15(5):e0232076.
