# Supplementary material for: COVID-19 pandemic partnership between medical students and isolated elders improves student understanding of older adults’ lived experience
Source: BMC Geriatr. 2022 Aug 2;22:636. doi: 10.1186/s12877-022-03312-z (PMC9344259; doi:10.1186/s12877-022-03312-z)
Supplement: Supplementary file 8 — Additional file 8. Lubben Social Network Scale Scores. [file 12877_2022_3312_MOESM8_ESM.docx]

**Additional file 8.** Lubben Social Network Scale Scores

| **Participant ID** | **Lubben Social Network Scale score** |
| --- | --- |
| 1 | 20 |
| 5 | 24 |
| 9 | 16 |
| 10 | 0 |
| 11 | 30 |
| 12 | 23 |
| 18 | 9 |
| 27 | 25 |
| 28 | 20 |
| 29 | 24 |
| 30 | 12 |
| 33 | 5 |
| 35 | 26 |
| 36 | 14 |
| 37 | 29 |
| 41 | 9 |
| 42 | 22 |
| 43 | 11 |
| 45 | 30 |
| 52 | 12 |
| 53 | 14 |
| 54 | 30 |
| 56 | 27 |
| 58 | 30 |
| 61 | 20 |
| 63 | 19 |
| 65 | 15 |
| 70 | 21 |
| 71 | 10 |
| 72 | 15 |
| 73 | 17 |
| 74 | 12 |
| 75 | 25 |
| 77 | 25 |
| 78 | 17 |
| 79 | 25 |
| 80 | 22 |
| 85 | 20 |
| 88 | 20 |
| 89 | 12 |
| 91 | 28 |
| 92 | 14 |
| 93 | 25 |
| 94 | 30 |
| 95 | 25 |
| 96 | 24 |
| 98 | 21 |

Lubben Social Network Scale Rankings

| **Scale ratings** | **Pre-intervention n=54** |
| --- | --- |
| 12 or lower = at risk | n = 11 (20%) |
| 13 or higher | n = 43 (80%) |

Scoring: None = 0; One = 1; Two = 2; Three or four = 3; Five thru eight = 4; Nine or more = 5

Total scale score is an equally weighted sum of the six items, with scores ranging from 0 to 30. 
A score of 12 and lower delineates “at-risk” for social isolation.

Source: Lubben J, Blozik E, Gillmann G, Iliffe S, Von Kruse WR, Beck JC, Stuck AE. Performance of an abbreviated version of the Lubben Social Network Scale among three European community-dwelling older adult populations. Gerontologist. 2006; 46: 503-513.
