# Supplementary material for: COVID-19 pandemic partnership between medical students and isolated elders improves student understanding of older adults’ lived experience
Source: BMC Geriatr. 2022 Aug 2;22:636. doi: 10.1186/s12877-022-03312-z (PMC9344259; doi:10.1186/s12877-022-03312-z)
Supplement: Supplementary file 9 — Additional file 9. UCLA Loneliness Scale and Warwick-Edinburgh Mental Well-being Scale Scores for Older Adults. [file 12877_2022_3312_MOESM9_ESM.docx]

**Additional file 9.** UCLA Loneliness Scale and Warwick-Edinburgh Mental Well-being Scale Scores for Older Adults

|  | **UCLA Loneliness Scale scores** | | **Warwick-Edinburgh Mental Well-being Scale scores** | |
| --- | --- | --- | --- | --- |
| **Participant ID** | **Pre-intervention** | **Post-intervention** | **Pre-intervention** | **Post-intervention** |

| 1 | 30 | 32 | 52 | 51 |
| --- | --- | --- | --- | --- |
| 5 | 48 | 39 | 49 | 52 |
| 9 | 42 | 47 | 48 | 53 |
| 10 | 75 | 70 | 49 | 37 |
| 11 | 20 | 23 | 70 | 70 |
| 12 | 25 | 20 | 65 | 65 |
| 18 | 53 | 65 | 40 | 32 |
| 27 | 29 | 39 | 53 | 46 |
| 28 | 37 | 24 | 59 | 60 |
| 29 | 53 | 51 | 42 | 38 |
| 30 | 42 | 45 | 46 | 47 |
| 33 | 66 | 60 | 56 | 51 |
| 35 | 27 | 58 | 54 | 38 |
| 36 | 36 | 51 | 35 | 36 |
| 37 | 28 | 38 | 54 | 56 |
| 41 | 36 | 41 | 54 | 62 |
| 42 | 41 | 35 | 53 | 49 |
| 43 | 50 | 38 | 62 | 56 |
| 45 | 25 | 22 | 66 | 67 |
| 52 | 53 | 38 | 51 | 46 |
| 53 | 31 | 33 | 66 | 60 |
| 54 | 31 | 25 | 59 | 55 |
| 56 | 47 | 47 | 46 | 50 |
| 58 | 32 | 23 | 54 | 67 |
| 61 | 47 | 42 | 50 | 52 |
| 63 | 55 | 52 | 39 | 48 |
| 65 | 41 | 29 | 50 | 50 |
| 70 | 42 | 38 | 55 | 60 |
| 71 | 65 | 55 | 57 | 54 |
| 72 | 20 | 40 | 53 | 58 |
| 73 | 57 | 48 | 51 | 57 |
| 74 | 43 | 48 | 46 | 47 |
| 75 | 20 | 24 | 60 | 57 |
| 77 | 21 | 22 | 48 | 65 |
| 78 | 42 | 48 | 45 | 54 |
| 79 | 36 | 39 | 54 | 50 |
| 80 | 45 | 48 | 46 | 45 |
| 85 | 67 | 69 | 41 | 43 |
| 88 | 21 | 23 | 59 | 52 |
| 89 | 20 | 20 | 58 | 59 |
| 91 | 29 | 36 | 46 | 42 |
| 92 | 42 | 35 | 52 | 51 |
| 93 | 51 | 47 | 48 | 50 |
| 94 | 36 | 27 | 59 | 58 |
| 95 | 40 | 28 | 50 | 57 |
| 96 | 20 | 20 | 57 | 51 |
| 98 | 21 | 23 | 69 | 64 |

UCLA Loneliness Scale Rankings

| **Scale ratings** | **Pre-intervention (n=47)** | **Post-intervention (n=47)** |
| --- | --- | --- |
| 20-34 = low | n= 18 (38.3%) | n=17 (36.2%) |
| 35-49 = moderate | n= 18 (38.3%) | n=21 (44.7%) |
| 50-64 = moderately high | n= 7 (15.0%) | n=6 (12.8%) |
| 65-80 = high | n= 4 (8.5%) | n=3 (6.4%) |

Scoring: Never = 1; Rarely =2; Sometimes = 3; Often = 4 ^*^Numbers may not equal 100 due to rounding

Source: https://fetzer.org/sites/default/files/images/stories/pdf/selfmeasures/Self_Measures_for_Loneliness_and_Interpersonal_Problems_UCLA_LONELINESS.pdf

Warwick-Edinburgh Mental Well-being Scale Rankings

| **Scale ratings** | **Pre-intervention (n=47)** | **Post-intervention (n=47)** |
| --- | --- | --- |
| 60 or more = high mental wellbeing | n= 7 (15.0%) | n=10 (21.3%) |
| 45-59 = average mental wellbeing | n= 35 (74.5%) | n=30 (63.9%) |
| 41-44 = possible depression | n= 2 (4.3%) | n=2 (4.3%) |
| 40 or less = probable depression | n= 3 (6.2%) | n=5 (10.6%) |

Scoring: None of the time = 1; Rarely =2; Some of the time = 3; Often = 4; All of the time = 5

^*^Numbers may not equal 100 due to rounding

Source: https://warwick.ac.uk/fac/sci/med/research/platform/wemwbs/using/howto
